# Trends of Platelet Indices in Acute Exacerbations of Chronic Obstructive Pulmonary Disease: A Longitudinal Follow-Up Study

**DOI:** 10.7759/cureus.115621

**Published:** 2026-09-01

**Authors:** Sivasubramanian M, Shanu S Igno, Nareshkumar B, Buvaneshwari Rajarethinam

**Affiliations:** 1 General Medicine, Sri Venkateswaraa University, Chennai, IND; 2 Community Medicine, Sri Venkateswaraa University, Chennai, IND

**Keywords:** acute exacerbation, chronic obstructive pulmonary disease, mean platelet volume, mechanical ventilation, platelet distribution width, platelet indices

## Abstract

Introduction

Platelet indices, including platelet distribution width (PDW), mean platelet volume (MPV), and platelet count, are readily available markers of platelet activation and systemic inflammation. This study evaluated their association with disease severity and clinical outcomes in patients with acute exacerbation of chronic obstructive pulmonary disease (AECOPD).

Methods

A prospective longitudinal follow-up study was conducted among 77 patients admitted with AECOPD at Kilpauk Medical College & Hospital, Chennai. PDW, MPV, and platelet count were assessed at admission and at two and four weeks. Patients were monitored for mechanical ventilation during hospitalization and subsequent hospital admissions. Associations were assessed using the chi-square test and independent t-test, with p<0.05 considered statistically significant.

Results

Of 77 participants, 63 (81.8%) were male subjects and 45 (58.4%) were aged 41-60 years. At admission, low PDW (<12%), MPV (<9 fL) and platelet count (<300,000/µL) were observed in 54 (70.1%), 59 (76.6%), and 56 (72.7%) participants, respectively. Nineteen subjects (24.7%) required mechanical ventilation and 21 (27.3%) had hospital admissions during follow-up. Higher PDW, MPV, and platelet count at admission were significantly associated with mechanical ventilation and hospital admissions (p=0.0005 for each). Persistently elevated PDW showed a highly significant association with the need for mechanical ventilation (χ² = 27.393, p=0.0005). Of the patients who required mechanical ventilation, 73.7% had persistently elevated PDW compared to only 12.1% among those who did not require ventilation.

Conclusion

Elevated and persistently elevated platelet indices, particularly PDW, MPV, and platelet count, were associated with mechanical ventilation and hospital admissions in patients with AECOPD. These routinely available parameters may help identify patients at higher risk of adverse outcomes.

## Introduction

Chronic Obstructive Pulmonary Disease (COPD) is among the most prevalent chronic respiratory disorders, with a global prevalence of approximately 10.1% among adults aged 40 years and above. It is the third leading cause of death worldwide, with nearly 90% of COPD-related deaths occurring in low- and middle-income countries [1]. It ranks among the top three global causes of death, accounting for 3.7 million fatalities annually, and its burden continues to rise in low- and middle-income countries [2]. COPD is a prevalent, preventable, and manageable respiratory disorder marked by persistent respiratory symptoms and irreversible airflow limitation, resulting from structural changes in the airways and/or alveoli, most commonly due to prolonged exposure to harmful particles or gases [3]. 

COPD is an inflammatory disease; it is understandable that many markers, such as C-reactive protein (CRP), tumour necrosis factor-alpha, interleukin (IL)-6, IL-8, fibrinogen, and neutrophil numbers and activation, are raised in patients with COPD [4]. Alterations in platelet activity are believed to play a significant pathophysiological role in a wide range of acute and chronic disease states, particularly those characterized by heightened local and/or systemic inflammation and thrombotic burden [5]. Several studies support the concept that platelets play an active role in the pathogenesis and progression of COPD, particularly through enhanced leukocyte elastase activity, increased formation of platelet-monocyte aggregates, and dysregulation of hypoxia-related signalling pathways [6-8].

Platelet activation in patients with COPD may occur through several mechanisms, including hypoxia, tachycardia, and hyperglycaemia. Once activated, platelets undergo morphological changes such as pseudopodia formation, which enhance platelet adhesion and aggregation. The presence of pseudopodia leads to heterogeneity in platelet size, reflected as an increase in platelet distribution width (PDW). Plateletcrit (PCT) represents the total circulating platelet mass and is considered an indicator of hypercoagulable states [9]. During episodes of acute exacerbation, the inflammatory burden in COPD intensifies, resulting in increased production of pro-inflammatory cytokines and acute-phase reactants, including IL-6, CRP, and fibrinogen. These inflammatory mediators may interfere with megakaryopoiesis, leading to the preferential release of smaller platelets from the bone marrow. Consequently, alteration in mean platelet volume (MPV) is often observed during acute exacerbations of COPD [10,11]. 

However, only a few studies have been done showing the relationship between MPV and the severity of COPD. Hence our study aimed to assess the association of platelet indices, including platelet distribution width, mean platelet volume, and platelet count, with the need for mechanical ventilation among patients with acute exacerbation of chronic obstructive pulmonary disease (AECOPD). Also, to assess whether persistently elevated platelet indices during follow-up are associated with recurrent hospital admissions among patients with AECOPD.

## Materials and methods

Study design and setting

This was a hospital-based prospective longitudinal follow-up study conducted in the Department of General Medicine, Kilpauk Medical College & Hospital, Chennai. The study was conducted over a recruitment period of six months, from 09/04/2021 to 09/10/2021. Each enrolled patient was followed prospectively for a period of six months from the date of enrolment, irrespective of the date of recruitment.

Study population and eligibility criteria

The study included all patients admitted with a primary diagnosis of acute exacerbation of COPD who had a prior confirmed diagnosis of COPD based on Forced Expiratory Volume in 1 second/Forced Vital Capacity (FEV1/FVC) <0.70. Patients with bronchial asthma, pneumothorax, pulmonary embolism, pulmonary edema, pulmonary tuberculosis, lung malignancies, or bronchiectasis were excluded. Patients with underlying COPD admitted with another primary diagnosis, such as stroke or acute myocardial infarction, and those who died during the study were also excluded.

Sample size and sampling technique

The sample size was calculated using the formula:

[n = \frac{4\sigma^2}{d^2}]

where σ is the standard deviation and d is the absolute precision.

In Mohapatra et al. [11], the mean and standard deviation of Platelet Distribution Width for acute exacerbation of COPD were reported as 11.8 ± 0.4437. With a standard deviation (σ) of 0.44 and absolute precision (d) of 0.1, the sample size was calculated as:

[n = \frac{4(0.44)^2}{(0.1)^2} = 77.44 \approx 77]

A total of 77 patients were included in the study using convenience sampling.

Data collection and study variables

Data collection was carried out using a pre-designed and pre-tested semi-structured proforma consisting of four components (Figure 1).

**Figure 1:**
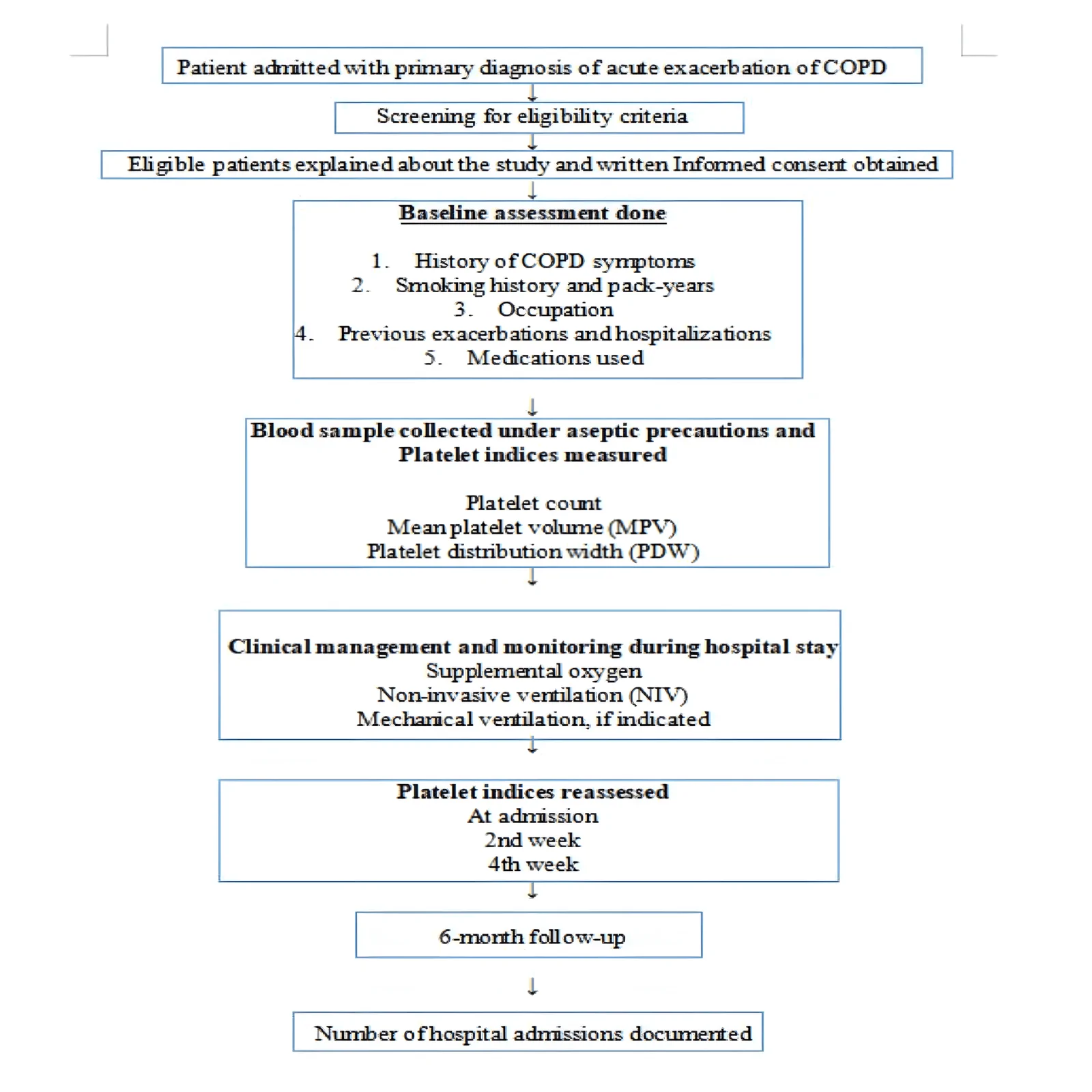
Data collection methodology

The first component documented clinical details and exposure history, including symptoms of COPD, smoking history with calculation of pack-years, occupational exposure, frequency of acute exacerbations, medications, and previous hospitalizations. The second component included laboratory assessment, wherein venous blood samples collected under strict aseptic precautions were used for estimation of platelet indices - MPV, PDW, and platelet count - using an automated hematology analyzer.The third component recorded in-hospital monitoring and management, including the requirement for supplemental oxygen, non-invasive ventilation, or mechanical ventilation as per standard clinical indications. The fourth component included follow-up and outcome assessment, with platelet indices measured at admission, at the second week, and at the fourth week. Patients were subsequently followed up for six months to document recurrent hospital admissions.

Measurement of platelet parameters

PDW, MPV, and platelet count were assessed at admission, and at two and four weeks. For analysis, PDW ≥12% was considered high and <12% low; MPV ≥9 fL was considered high and <9 fL low; and platelet count ≥3 lakhs/µL was considered high and <3 lakhs/µL low.

Persistent elevation was assessed during the follow-up measurements.

Study outcomes

The clinical outcomes assessed were the requirement for mechanical ventilation during hospitalization and hospital admissions during the six-month follow-up period. Recurrent hospital admission was defined as two or more hospital admissions during the six-month follow-up period.

Statistical analysis

The data collected were entered in Microsoft Excel 2019 (Microsoft Corp., Redmond, WA, USA) and analyzed using IBM SPSS Statistics for Windows, Version 23 (Released 2015; IBM Corp., Armonk, New York, United States). Quantitative data were expressed as mean, range, frequency, and distribution. The independent t-test and chi-square test were used to assess associations. A p-value <0.05 was considered statistically significant. Pie charts and bar graphs were used to represent the data pictorially.

Ethical considerations

After obtaining approval from the Scientific Research Committee and Institutional Human Ethical Committee, the study was conducted in the Department of General Medicine, Kilpauk Medical College & Hospital, Chennai. The study was approved by the Institutional Human Ethical Committee (IEC), Kilpauk Medical College & Hospital, Chennai (approval no. 530/2021; dated 08/04/2021).

## Results

A total of 77 participants were included in the study. The majority of participants belonged to the 41-60 years age group, accounting for 45 (58.40%) of the total study population. This was followed by the 61-80 years age group with 28 (36.40%) participants. The least represented group was 21-40 years, comprising only four (5.20%) participants. This indicates that most study participants were middle-aged to elderly individuals, with a predominance of subjects in the 41-60 years age category (Figure 2).

**Figure 2:**
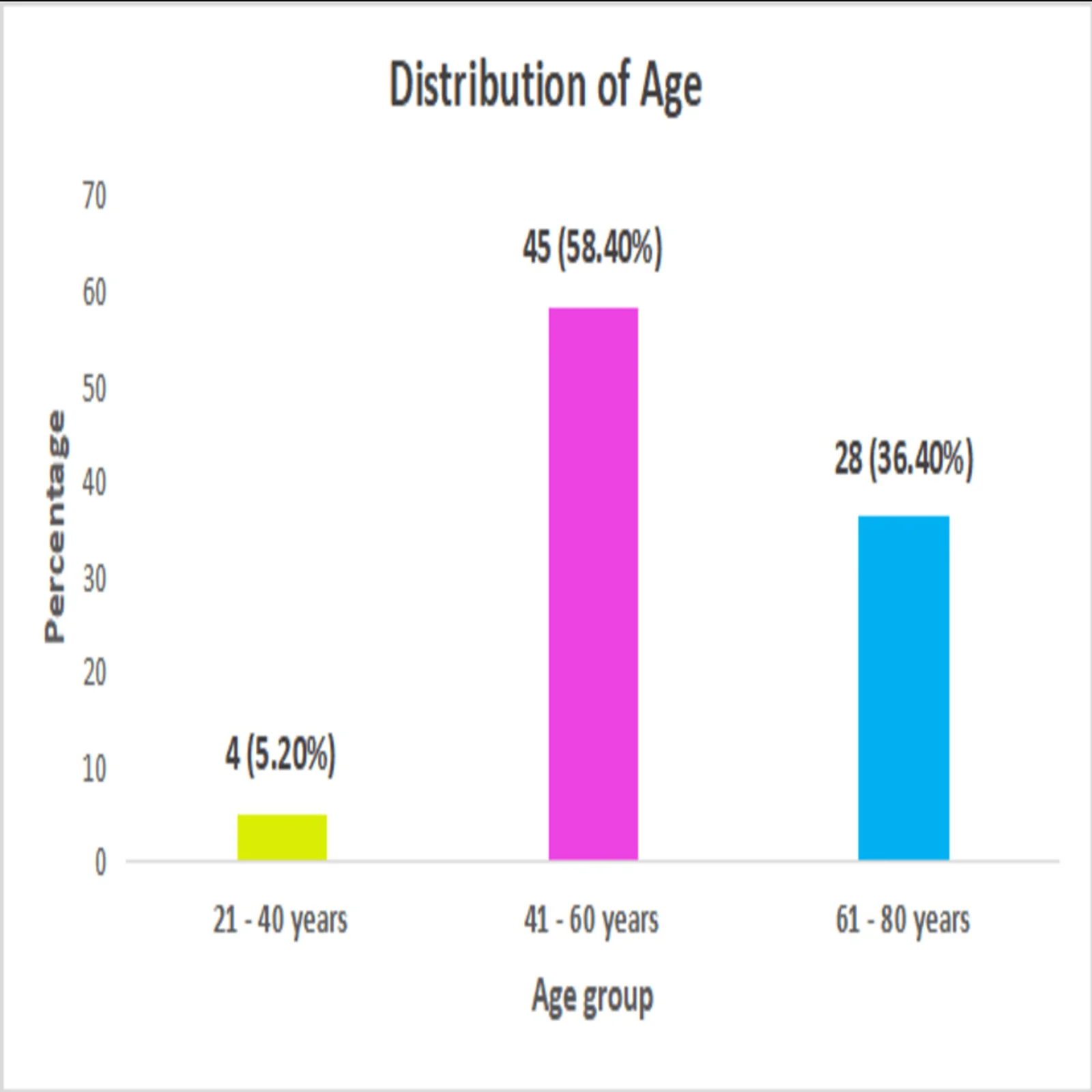
Frequency distribution of age among the participants (N=77)

Among the total study population, the majority were male subjects, comprising 63 (81.80%) participants, while female subjects accounted for 14 (18.20%) participants (Figure 3).

**Figure 3:**
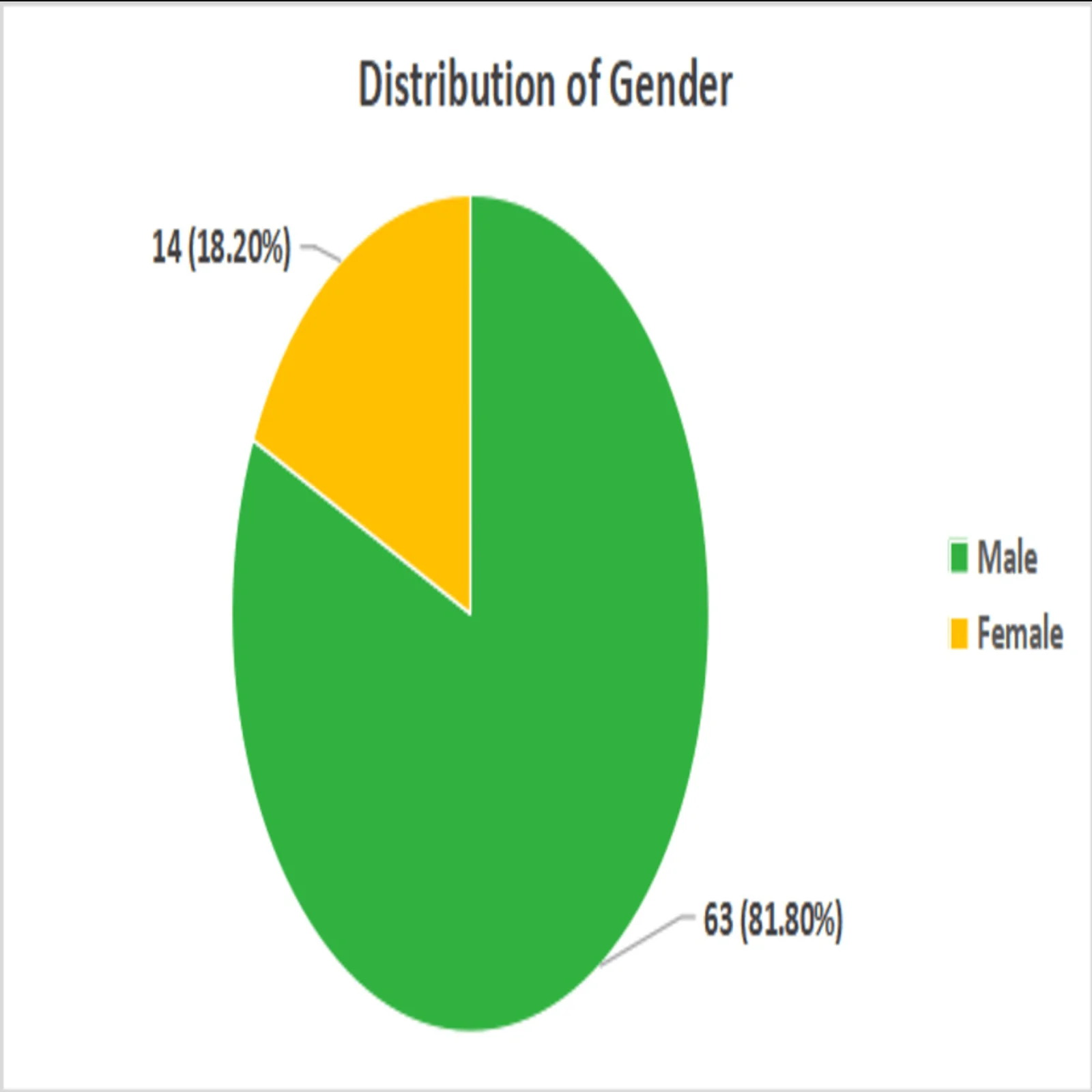
Gender distribution among the study participants (N=77)

The majority of participants had no comorbidities, accounting for 59 (76.60%), while 18 (23.40%) participants had associated comorbid conditions (Table 1).

**Table 1:** Baseline characteristics of the study participants (N=77) PDW, platelet distribution width; MPV, mean platelet volume.

Variable	Category	Frequency	Percentage
Comorbidity	Absent	59	76.60%
	Present	18	23.40%
PDW at admission	Low	54	70.10%
	High	23	29.90%
MPV at admission	Low	59	76.60%
	High	18	23.40%
Platelet count at admission	Low	56	72.70%
	High	21	27.30%

Regarding platelet indices at admission, low PDW (<12%) was observed in 54 (70.10%) participants, whereas 23 (29.90%) had high PDW values (≥12%). Similarly, low MPV (<9 fL) was noted in 59 (76.60%) participants, while 18 (23.40%) had high MPV values (≥9 fL). For platelet count at admission, 56 (72.70%) participants had low platelet counts (<3 lakhs/µL), while 21 (27.30%) had high platelet counts (≥3 lakhs/µL) (Table 1).

Persistently high PDW was observed in 21 (27.30%) participants, while 56 (72.70%) did not show persistently elevated PDW values. Similarly, persistently high MPV was noted in 19 (24.70%) participants, whereas 58 (75.30%) had no persistent elevation. Persistently high platelet count was identified in 21 (27.30%) participants, while 56 (72.70%) did not have persistent elevation. During follow-up, 19 (24.70%) participants required mechanical ventilation, while 58 (75.30%) did not require ventilatory support. Additionally, 21 (27.30%) participants had hospital admissions, whereas 56 (72.70%) did not require hospital admission (Table 2).

**Table 2:** Frequency distribution of persistently high platelet parameters and outcomes among the study participants (N=77) PDW, platelet distribution width; MPV, mean platelet volume; High PDW, ≥12%; High MPV, ≥9 fL; High platelet count, ≥3 lakhs/µL.

Variable	Yes	No
PDW Persistently High	21 (27.30%)	56 (72.70%)
MPV Persistently High	19 (24.70%)	58 (75.30%)
Platelet Count Persistently High	21 (27.30%)	56 (72.70%)
Mechanical Ventilation	19 (24.70%)	58 (75.30%)
Hospital Admissions	21 (27.30%)	56 (72.70%)

Participants with high PDW at admission showed a greater requirement for mechanical ventilation (n=17; 89.50%) compared to those with low PDW (n=2; 10.50%), and this association was found to be highly statistically significant (χ² = 42.778, p=0.0005). Similarly, participants with high MPV at admission had a higher requirement for mechanical ventilation (n=12; 63.20%) compared to those with low MPV (n=7; 36.80%), showing a statistically significant association (χ² = 22.286, p=0.0005). Participants with high platelet count at admission also demonstrated an increased need for mechanical ventilation (n=14; 73.70%) compared to those with low platelet count (n=5; 26.30%), which was statistically significant (χ² = 27.393, p=0.0005).

Persistently elevated platelet parameters were also significantly associated with mechanical ventilation. Participants with persistently high PDW (n=14; 73.70%), persistently high MPV (n=12; 63.20%), and persistently high platelet count (n=14; 73.70%) had a higher likelihood of requiring mechanical ventilation compared to those without persistent elevation. All these associations were found to be highly statistically significant (p=0.0005) (Table 3).

**Table 3:** Association of platelet parameters with mechanical ventilation among the participants (N=77) p-value <0.05 is statistically significant.

Variable	Category	Mechanical Ventilation		Chi square (p-value)
		Yes n (%)	No n (%)	
Platelet Distribution Width at Admission	Low	2 (10.50%)	52 (89.70%)	42.778 (0.0005)
	High	17 (89.50%)	6 (10.30%)	
Mean Platelet Volume at Admission	Low	7 (36.80%)	52 (89.70%)	22.286 (0.0005)
	High	12 (63.20%)	6 (10.30%)	
Platelet Count at Admission	Low	5 (26.30%)	51 (87.90%)	27.393 (0.0005)
	High	14 (73.70%)	7 (12.10%)	
Persistently High Platelet Distribution Width	Yes	14 (73.70%)	7 (12.10%)	27.393 (0.0005)
	No	5 (26.30%)	51 (87.90%)	
Persistently High Mean Platelet Volume	Yes	12 (63.20%)	7 (12.10%)	20.098 (0.0005)
	No	7 (36.80%)	51 (87.90%)	
Persistently High Platelet Count	Yes	14 (73.70%)	7 (12.10%)	27.393 (0.0005)
	No	5 (26.30%)	51 (87.90%)	

Participants with high PDW at admission had a significantly higher proportion of hospital admissions (n=18; 85.70%) compared to those with low PDW (n=3; 14.30%). This association was highly statistically significant (χ² = 29.575, p=0.0005). Likewise, participants with high MPV at admission demonstrated a higher frequency of hospital admissions (n=16; 76.20%) compared to those with low MPV (n=5; 23.80%), showing a statistically significant association (χ² = 30.210, p=0.0005). A similar trend was observed with platelet count at admission, where participants with high platelet count had increased hospital admissions (n=17; 81.00%) compared to those with low platelet count (n=4; 19.00%), and this association was statistically significant (χ²=34.836, p=0.0005).

Persistent elevation of platelet parameters was also significantly associated with hospital admissions. Participants with persistently high PDW and persistently high platelet count had hospital admission rates of 81.0%, while those with persistently high MPV showed the highest hospital admission proportion (85.7%). All these associations were found to be highly statistically significant (p<0.01) (Table 4).

**Table 4:** Association of platelet parameters with hospital admissions among the study participants (N=77) p-value <0.05 is statistically significant.

Variable	Category	Hospital Admissions		Chi square (p-value)
		Yes n (%)	No n (%)	
Platelet Distribution Width at Admission	Low	3 (14.3%)	51 (91.1%)	29.575 (0.0005)
	High	18 (85.7%)	5 (8.9%)	
Mean Platelet Volume at Admission	Low	5 (23.8%)	54 (96.4%)	30.210 (0.0005)
	High	16 (76.2%)	2 (3.6%)	
Platelet Count at Admission	Low	4 (19.0%)	52 (92.9%)	34.836 (0.0005)
	High	17 (81.0%)	4 (7.1%)	
Persistently High Platelet Distribution Width	Yes	17 (81.0%)	4 (7.1%)	34.836 (0.0005)
	No	4 (19.0%)	52 (92.9%)	
Persistently High Mean Platelet Volume	Yes	18 (85.7%)	1 (1.8%)	57.881 (0.0005)
	No	3 (14.3%)	55 (98.2%)	
Persistently High Platelet Count	Yes	17 (81.0%)	4 (7.1%)	34.836 (0.0005)
	No	4 (19.0%)	52 (92.9%)	

Participants requiring mechanical ventilation had consistently higher PDW values at admission, and at two and four weeks compared to those not requiring ventilation. These differences were found to be statistically significant, with highly significant associations at admission and two weeks (p<0.001) and significant association at four weeks (p=0.010). Similarly, MPV was higher among participants requiring mechanical ventilation at admission and two weeks, showing statistically significant differences (p=0.042 and p<0.001, respectively). However, at four weeks, the difference in MPV between the two groups was not statistically significant (p=0.846). Likewise, platelet count was significantly higher among participants requiring mechanical ventilation at admission, and at two and four weeks compared to those not requiring ventilation, with highly statistically significant differences (p<0.001) (Table 5).

**Table 5:** Comparison of platelet parameters with mechanical ventilation by unpaired sample t-test (N=77) p-value <0.05 is statistically significant.

Variable	Time point	Mechanical Ventilation	N	Mean	SD	t-value (p-value)
Platelet Distribution Width	Admission	Yes	19	13.7	1.1	9.029 (<0.001)
		No	58	10.7	1.3	
	2 weeks	Yes	19	13.3	1.6	6.731 (<0.001)
		No	58	10.6	1.1	
	4 weeks	Yes	19	12.3	2.0	2.816 (0.010)
		No	58	10.9	1.2	
Mean Platelet Volume	Admission	Yes	19	9.7	1.8	2.163 (0.042)
		No	58	8.8	1.0	
	2 weeks	Yes	19	10.0	1.7	3.150 (<0.001)
		No	58	8.7	0.9	
	4 weeks	Yes	19	8.7	1.3	0.195 (0.846)
		No	58	8.6	1.3	
Platelet Count	Admission	Yes	19	3.6	0.9	5.943 (<0.001)
		No	58	2.3	0.8	
	2 weeks	Yes	19	3.3	0.9	4.365 (<0.001)
		No	58	2.3	0.9	
	4 weeks	Yes	19	3.1	0.9	0.553 (<0.001)
		No	58	2.0	0.7	

Participants who required hospital admission had consistently higher PDW values at admission, and at two and four weeks compared to those without hospital admission. These differences were found to be highly statistically significant at all time points (p<0.001). Similarly, MPV was higher among participants requiring hospital admission at admission and at two weeks, with statistically significant differences observed (p=0.004 and p <0.001, respectively). However, at four weeks, the difference in MPV between admitted and non-admitted participants was not statistically significant (p=0.109). Likewise, platelet count was significantly higher among participants requiring hospital admission at admission, and at two and four weeks compared to those without hospital admission, with all differences being highly statistically significant (p<0.001) (Table 6).

**Table 6:** Comparison of platelet parameters with hospital admissions by unpaired sample t-test (N=77) p-value <0.05 is statistically significant.

Variable	Time point	Hospital Admission	N	Mean	SD	t-value (p-value)
Platelet Distribution Width	Admission	Yes	21	13.1	1.5	6.249 (<0.001)
		No	56	10.8	1.4	
	2 weeks	Yes	21	13.6	1.3	10.580 (<0.001)
		No	56	10.4	0.8	
	4 weeks	Yes	21	13.0	1.8	6.070 (<0.001)
		No	56	10.6	0.8	
Mean Platelet Volume	Admission	Yes	21	10.0	1.9	3.207 (0.004)
		No	56	8.6	0.6	
	2 weeks	Yes	21	10.2	1.5	4.897 (<0.001)
		No	56	8.5	0.8	
	4 weeks	Yes	21	9.1	1.4	1.647 (0.109)
		No	56	8.5	1.2	
Platelet Count	Admission	Yes	21	3.6	0.9	7.012 (<0.001)
		No	56	2.2	0.7	
	2 weeks	Yes	21	3.7	0.9	8.535 (<0.001)
		No	56	2.2	0.6	
	4 weeks	Yes	21	3.1	0.8	5.976 (<0.001)
		No	56	2.0	0.6	

## Discussion

MPV is a routinely measured hematological parameter that reflects platelet size and activation status. Increased platelet activation has been implicated in inflammatory and thrombotic disorders and is believed to contribute to the systemic inflammatory milieu observed in COPD. In COPD, the degree of systemic inflammation has been shown to correlate with disease severity, as classified by the Global Initiative for Chronic Obstructive Lung Disease (GOLD) criteria. In addition, platelet activation may also contribute to the increased cardiovascular risk frequently observed as a comorbidity in COPD patients. Previous studies have evaluated the role of MPV as a potential inflammatory biomarker in COPD and other chronic inflammatory conditions, including cardiovascular disease, cerebrovascular disorders, rheumatoid arthritis, and diabetes mellitus. However, findings regarding the association between MPV and COPD have been inconsistent across studies, highlighting the need for further research to better understand the platelet indices in COPD, particularly during acute exacerbations [12].

In the present study, the majority of participants belonged to the 41-60 years age group (58.4%), followed by 61-80 years (36.4%), while only 5.2% were aged 21-40 years. This indicates that AECOPD predominantly affected middle-aged and elderly individuals in the study population.

This finding is consistent with the natural history of COPD, which is more common among older adults due to cumulative exposure to smoking, biomass fuel, occupational pollutants, and progressive decline in lung function. Similar age predominance was reported by Ali et al., where the majority of COPD patients were above 50 years of age [10]. Archita and Ashok also observed that AECOPD was more common in older adults, reflecting the chronic progressive nature of the disease [9].

In this study, male subjects constituted 81.8% of participants, while female subjects accounted for 18.2%. The predominance of male subjects may be explained by higher rates of tobacco smoking, occupational dust exposure, and outdoor pollutant exposure among men. This observation is in agreement with Ali et al. and Archita and Ashok [9,10], both of whom reported male predominance among COPD patients. Although COPD prevalence in women is increasing globally due to biomass exposure and smoking trends, male predominance continues to be observed in many Indian hospital-based studies [9,10].

At admission, 70.1% of participants had low PDW, 76.6% had low MPV, and 72.7% had low platelet count. These findings suggest that although baseline platelet parameters may be within lower ranges in many patients, a subgroup with elevated indices may represent heightened inflammatory or prothrombotic activity. Ulasli et al. demonstrated significant alteration in MPV during acute exacerbations, supporting its role as an inflammatory biomarker [13]. Similarly, Steiropoulos et al. found altered MPV and PDW levels in COPD patients, suggesting platelet activation secondary to systemic inflammation and hypoxia [14].

Persistently elevated values were observed in patients: PDW (27.3%), MPV (24.7%) and platelet count (27.3%). Persistent elevation of platelet indices likely reflects ongoing systemic inflammation and unresolved disease activity. Most previous studies were cross-sectional, whereas the present study uniquely followed patients longitudinally over four weeks. This longitudinal finding adds important evidence that sustained platelet activation may be associated with poor recovery. Zinellu et al. in their systematic review noted heterogeneity in platelet indices across COPD studies and emphasized the need for longitudinal data [5]. Thus, the current study provides novel insight into the persistence of platelet abnormalities after acute exacerbation.

Association with mechanical ventilation

High platelet parameters at admission were significantly associated with increased need for mechanical ventilation. Patients with high PDW had significantly higher mechanical ventilation requirement (89.5%, p=0.0005). PDW reflects variability in platelet size and is considered a marker of platelet activation and inflammatory stress by Vagdatli et al [15]. Patients with elevated MPV had significantly higher ventilation requirement (63.2%, p=0.0005). Ulasli et al. demonstrated significant changes in MPV during acute exacerbations, supporting its potential role as an inflammatory biomarker in COPD. High platelet count was significantly associated with ventilation need (73.7%, p=0.0005). Reactive thrombocytosis may reflect acute inflammation mediated by cytokines such as IL-6 [13]. 

Association with hospital admission

Patients with elevated platelet indices had significantly higher hospital admissions: high PDW (85.7%), high MPV (76.2%) and high platelet count (81.0%); and all associations were statistically significant. These findings indicate that platelet activation markers may help identify disease severity requiring inpatient care. Muñoz-Esquerre et al. demonstrated increased platelet reactivity during acute exacerbations, suggesting an association between platelet activation and disease severity. Ramya et al. also reported altered hematological biomarkers in AECOPD patients [7,16].

Patients requiring mechanical ventilation had significantly higher PDW values at admission, and at two and four weeks. This persistent significance suggests PDW may be a robust longitudinal marker. MPV was higher at admission and two weeks but not significant at 4 weeks. This suggests MPV may reflect acute inflammatory burden but normalizes with recovery. Similar alterations in MPV during acute exacerbation have been reported by Ali et al. [10] and Ulasli et al. [13], supporting the role of MPV as an inflammatory marker in COPD. Platelet count was higher across all time points. This indicates sustained thrombopoietic or inflammatory response in severe disease.

Patients requiring hospital admission demonstrated significantly higher PDW and platelet count at all time points, whereas MPV was significantly elevated only at admission and at two weeks. The persistent significance of PDW and platelet count suggests their being superior compared with MPV. Zinellu et al. [5], in a systematic review and meta-analysis, highlighted the potential clinical utility of platelet indices in COPD while emphasizing heterogeneity across studies and the need for further validation.

Limitations

This study was conducted at a single tertiary care center with a relatively small sample size, particularly among patients requiring mechanical ventilation, which may limit generalizability. Platelet indices may be influenced by comorbidities, medications, infection severity, and other confounding factors. Categorizing platelet indices into high and low groups may have resulted in some loss of information. As an observational study with a four-week follow-up, the findings indicate association rather than causation and do not establish long-term prognostic value.

## Conclusions

Platelet indices may have potential utility as simple, accessible, and cost-effective markers for assessing disease severity and identifying patients with COPD who are at increased risk of adverse clinical outcomes. Their routine assessment may provide additional information for clinical risk stratification alongside established clinical and physiological parameters. Monitoring changes in platelet indices over the course of illness may also assist clinicians in recognizing patients who require closer observation and timely management. Incorporating these readily available hematological parameters into clinical assessment may therefore have potential value, particularly in resource-limited healthcare settings. However, platelet indices should be considered as complementary markers rather than substitutes for comprehensive clinical evaluation and established measures of COPD severity. Further prospective studies with larger sample sizes, standardized thresholds, longer follow-up, and appropriate adjustment for potential confounding factors are warranted to establish their independent prognostic and predictive value in COPD.
